# The view and policy of management of occupational health services on the performance of workers’ health surveillance: a qualitative exploration

**DOI:** 10.1186/s12913-019-4296-6

**Published:** 2019-07-10

**Authors:** Felicia S. Los, Carel T. J. Hulshof, Judith K. Sluiter

**Affiliations:** Coronel Institute of Occupational Health, Amsterdam UMC, Academic Medical Center, PO Box 22700, 1100 DE Amsterdam, The Netherlands

**Keywords:** Workers’ health surveillance, Occupational health service, Occupational physician, Management

## Abstract

**Background:**

Although workers’ health surveillance is an important preventive activity, it is not regularly performed. In addition to the occupational physician, the management of occupational health services can also be involved in the performance of workers’ health surveillance. The present study investigated the view and policy of the managements of occupational health services on the performance of workers’ health surveillance by occupational physicians.

**Method:**

Semi-structured face-to-face interviews about the mission, view, and policy of the occupational health services with respect to workers’ health surveillance were conducted with eighteen randomly selected board members of occupational health services in the Netherlands. The results were transcribed verbatim and were analysed using MAXQDA software to form themes and categories.

**Results:**

The first theme found was the view of the management of occupational health services. Categories found were mission statements of occupational health services and the attitude of the management of occupational health services towards workers’ health surveillance. Three types of mission statements were mentioned by the board members: keeping workers at work, improving the health of workers, or helping the employer with sick-leave management. Both positive and negative attitudes towards workers’ health surveillance appeared from the interviews. Some board members mentioned that workers’ health surveillance can improve workers’ health, and creates awareness about workers’ health. Other board members mentioned that performing workers’ health surveillance is eliciting problems, and that employers do not have a positive attitude towards workers’ health surveillance. The second theme was the policy on performing workers’ health surveillance. Categories found were the policy on performing workers’ health surveillance towards companies, and the policy on performing workers’ health surveillance towards professionals. Some occupational health services recommend workers’ health surveillance to all companies. However, in general workers’ health surveillance was only performed at request of companies, and no instructions or training programmes for occupational physicians were provided.

**Conclusion:**

Although some of the mentioned views on workers’ health surveillance are positive, the policy of occupational health services on workers’ health surveillance does, so far, in general, not stimulate occupational physicians or employers to perform or organize workers’ health surveillance.

## Background

Workers’ health surveillance (WHS) includes the assessment of workers’ health and factors in the working environment that can have a negative influence on the health and wellbeing of workers [1]. WHS is aimed at the prevention of work-related diseases, and the maintenance and promotion of workers’ health. It is an important activity in occupational health care, developed to protect workers in the new and rapidly changing environment [1–3].

In the Netherlands, a guidance document was developed by the Netherlands Society of Occupational Medicine to support the occupational physician (OP) with the performance of WHS [2] . According to this guidance document, three core objectives of WHS can be distinguished 1) the prevention of work-related diseases, 2) monitoring and promoting workers’ work-related health, and 3) maintaining and improving workers’ health and sustainable employability of workers [2]. Employers are legally obliged to periodically offer workers a WHS aimed at at least the first core objective; the prevention of work-related diseases [4]. Several studies have found that WHS, when carried out according to the guidance document, can prevent work-related diseases, improve work functioning, and can have financial benefits for employers [5–7].

OPs are employed at an occupational health service (OHS) or are self-employed [8]. To comply to the legal obligation to periodically offer WHS to workers, companies can hire an OHS or a specific expert, an OP [9]. In a study by Moriguchi et al., it was concluded that, compared to their Japanese counterparts, Dutch OPs spend a large amount of time managing workers’ sickness absence and little time on preventing workers’ health problems [10]. Assuming that WHS is such a preventive activity, this means that despite the legal obligation for employers [4], WHS is not regularly performed.

In promoting and implementing WHS, the behaviour of the OP is crucial. Several factors influence this. A model that can be used to develop and implement integrated health policies is the behaviour change ball (BCB) [11]. According to the BCB model, the behaviour of several actors at different organizational levels can influence the implementation and performance of a health policy [11]. Applied to the performance of WHS, this means that besides the behaviour of the OP, other stakeholders can also have an influence on the performance of WHS. The BCB model distinguishes three levels within an organization at which stakeholders can act: 1) the strategic level, where actors are involved in decisions concerning what will be done and define the short-term goals for the organization; 2) the tactical level, where actors are involved in decisions concerning how it will be done; and 3) the operational level, where actors are involved in the actual implementation of the policy [11, 12].

In order to improve the performance of WHS, actors at different organizational levels are important. The aim of the present study was to investigate the view and policy on the performance of WHS of an acting party at the strategic level, namely the management of the OHS. The research questions were:

1. What is the view of the management of the OHS on WHS?
2. What is the current policy on WHS within the OHS?

## Methods

### Context and study design

The context of the study is the occupational health care system in the Netherlands. The present study is an explorative qualitative study. The reporting of the study is based on the ‘Consolidated criteria for reporting qualitative research’ [13].

### Sampling strategy

Board members from OHSs in the Netherlands were interviewed. At the moment of recruiting (May, 2017), 83 external (i.e. independent) OHSs and 21 internal (i.e. company) OHSs were registered in the Netherlands [14]. Thirty OHSs were randomly selected using a random number generator [15]. Board members of the selected OHSs received an information letter and invitation by letter or email. If a board member had not rejected the invitation within 2 weeks after sending the invitation, FL telephoned the OHS to make an appointment with the board member for an interview.

### Data collection

Interview questions were developed based on the guidance document on WHS, [2] and the BCB model [11]. An interview guide was used to conduct the semi-structed interviews with managers of OHSs (Appendix). The interviews were semi-structured and consisted of open questions about the mission of the OHS, and view and policy of the management of the OHS on promoting and implementing WHS. The use of open questions allowed the researcher to gain deeper insight into the participants’ answers by asking follow-up questions. The interviews were conducted by FL.

To answer the first research question, the interviewees were asked:

- What is the mission statement of the OHS?
- What is the view of the OHS on WHS?

To answer the second research question, the interviewees were asked:

- What is the current strategy of the OHS regarding WHS?
- What is the current policy of the OHS on WHS?

All interviews were recorded with an audio-recorder.

### Data processing

All interviews were transcribed verbatim. Interviews were registered using a unique code for each interview, ensuring that the data were processed anonymously.

### Data analysis

Interviews were analysed using thematic analysis [16]. The data was managed using MAXQDA software (Verbi GmbH, Marburg, Germany). All three researchers first familiarized themselves with the data by reading the transcripts multiple times. A list with initial codes from the obtained answers was then drawn up and data-driven codes were generated. After the coding process, discussions were held in the project group. Themes were then formulated by FL and CH to answer the research questions. Finally data saturation was reached, and conclusions were drawn.

## Results

**Fig. 1:**
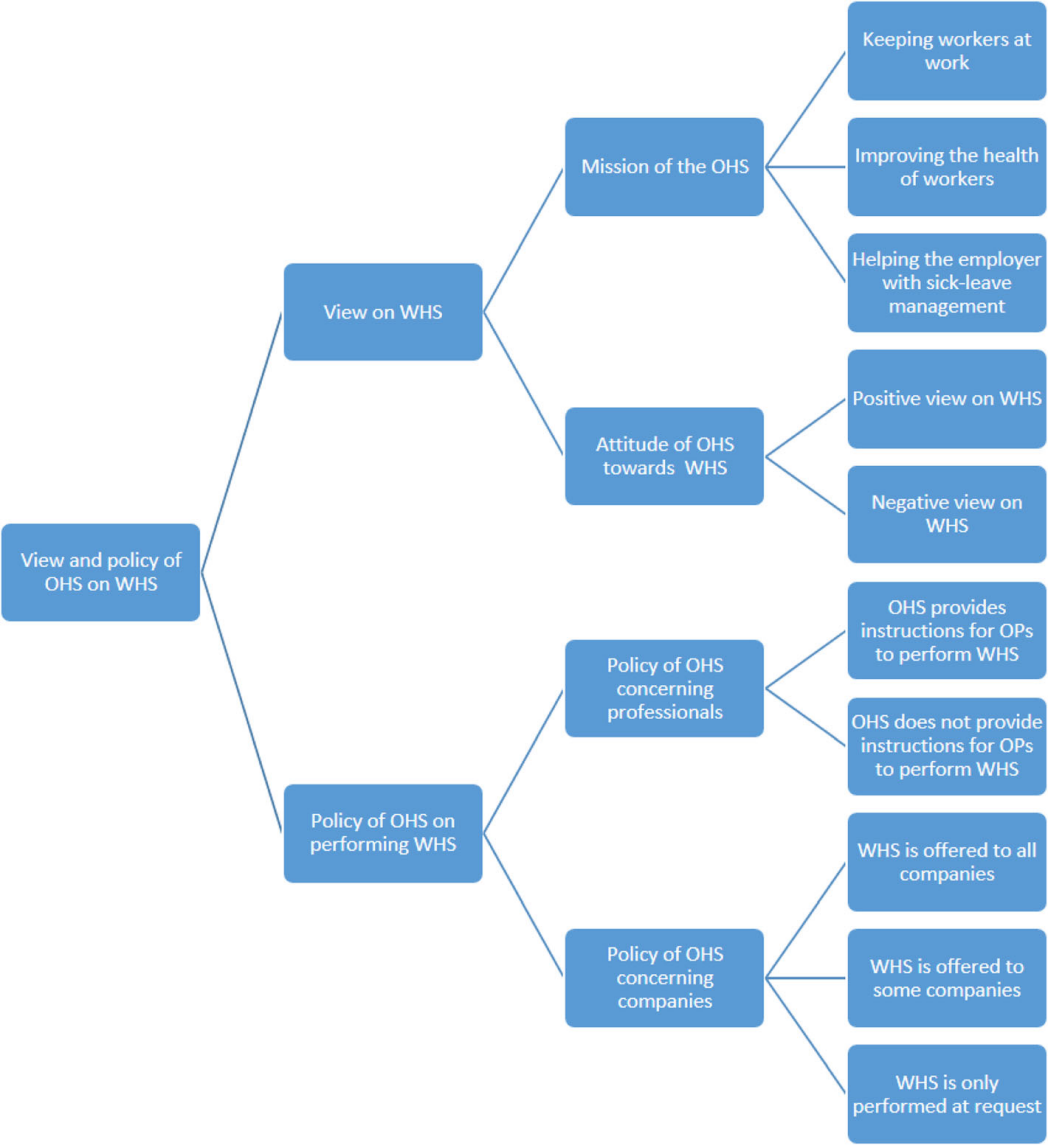
code tree

In the period June–August 2017, we conducted nineteen interviews with board members of OHSs in the Netherlands. Interviews were conducted at the OHS. Duration of the interviews was about 30–60 min. The size of the OHSs varied from one that had only one operating OP, to one with 400 OPs. Because one of the OHSs in the sample positions itself rather as a sector institute instead of an OHS, this interview was not included in the results. Results are described by two main themes: the view of the management of the OHS on WHS, and the current policy of the OHS on the performance of WHS. Categories, and subcategories were distinguished (see Fig. 1: code tree).

### The view on WHS

#### The mission of the OHS

Three types of mission statements were reported by managers of OHSs. Some managers formulated their mission as helping the employer with sick-leave management. Other managers reported that the mission statements of their OHSs are focussed on keeping workers at work or improving the health of workers.

- Keeping workers at work

*‘So really the mission is to keep workers at work as well, as healthy and for as long as possible (interviewee 5)’.*

*‘Keep the worker employable (interviewee 17)’*

b. Improving the health of workers

*‘The mission of our occupational health and safety service is to improve the health, safety and well-being of workers at a company as much as possible (interviewee 11)’.*

*‘Working on the health of workers (‘interviewee 18)’*

c. Helping the employer with sick-leave management

*‘The mission is very clearly intended to reduce absenteeism from a consensus of providing very broad support to the employer (4) ’.*

*‘We want to motivate and to inspire people to come to inner awareness. To make them aware of the fact that you have to start anticipating earlier on sick-leave (interviewee 8)’.*

#### Attitude of the management of the OHS towards WHS

Positive and negative views on WHS emerged from the interviews. In general, board members who were positive about WHS stated that the added value of WHS is about workers’ health.

- Positive views on WHS

*‘We think it is a nice tool for the implementation of workers’ health promotion activities (interviewee 5)’.*

*‘I think that WHS can create awareness (interviewee 10)'.*

b. Negative views on WHS

Negative views on WHS emerged mostly from difficulties with implementing WHS, such as a negative attitude of employers towards WHS, and the time needed to perform WHS.

*‘It is more a fear of our OPs that if we start offering WHS to all our workers on the intranet, it will be impossible to plan because the physicians’ diaries are already so full (interviewee 6)’.*

*‘I have never seen anyone enthusiastic about WHS (interviewee 2)’.*

*‘There are also costs involved, not everyone wants to invest (interviewee 17)’.*

However, some board members also mentioned they are not convinced of the added value of WHS.

*‘If you zoom into something closely enough, you will always find someone with complaints (interviewee 2)’.*

*‘I think at the moment [..] there are complaints but not yet absenteeism, so we’re still at the prevention stage, then you can achieve more I think with a number of conversations (interviewee 16)’.*

*‘It is increasingly less relevant and often just a thing that has to be done, so it doesn’t lead to relevant changes (interviewee 15)’.*

### The policy of OHSs on the performance of WHS

#### The policy on WHS towards professionals

As WHS should be performed by an OP, instructions or training for the performance of WHS can be part of the policy of an OHS. Board members were asked if they provide any instructions, training or other forms of support to the OP in relation to carrying out WHS. In some situations, the managements of OHSs do provide instructions for WHS, for example when the OHSs is an educational institute for OPs in training. However, the majority of the board members did not provide any support to the OP in relation to carrying out WHS. Reasons mentioned for not doing so were that WHS is OPs’ responsibility, or that WHS is not performed at all at the OHS.

- Instructions about implementing WHS are provided by the OHS:

*‘As soon as we do something different than usual, then we have to give an instruction about that’ (interviewee 14)’.*

*‘We are a workplace educational institute, so we train the junior OPs, also in WHS (interviewee 18) ’*

b. Instructions about implementing WHS are not provided by the OHS:

*‘No instructions are provided. It is the responsibility of the OP to keep up-to-date (interviewee 5) ’.*

‘*No. Yes there was a doctor who did this a few times. But now he always does it, so when there is a request for WHS, it ends up on his desk (interviewee 16) ’.*

‘*We don’t offer a WHS program that is meeting the recommendations of Netherlands Society of Occupational Medicine (NVAB) (interviewee 9)’.*

#### The policy on WHS towards companies

In general, two strategies are used to start a WHS programme. The first is to recommend WHS to the companies (i.e. employer or works council), as indicated. The second is a passive strategy, namely to perform WHS only at the request of the company (i.e. employer). Board members had adopted this strategy, because their customers, in particular employers, were not positive about WHS. Other OHSs offered WHS to some of the companies.

- The OHS recommends WHS to the company (i.e. employer or works council):

‘*We always advise the employer to perform WHS (interviewee 7)’*

b. WHS is only offered to some companies:

*‘ Our experience is that smaller companies are not looking for WHS. We have an annual meeting with larger companies to discuss the situation (interviewee 4)’.*

*‘With large companies, we make an annual plan every year, in which we advice on the approach to sickness absence management and the preventive measures that can be taken (interviewee 12)’*

c. WHS is only performed at request of the company (i.e. employer):

*‘At request of the company – but also because many companies use us for sickness absence management rather than prevention, but until now our customers did not see the added value of WHS, just that it costs a lot of money (interviewee 16)’.*

*‘ We are not actively offering WHS, that has to do with the fact that I do not see the added value of WHS (interviewee 2)’.*

## Discussion

In answer to the first research question (‘What is the view of the management of OHSs on WHS?’), we found that a substantial part of the board members have a negative view on WHS. Other board members have a more positive view, they are convinced of the added value of WHS. Regarding the second research question (‘What is the current policy on WHS within the OHS?’), we found that in general board members do not provide training or instructions or any other form of support in carrying out WHS. The majority of the board members mentioned they only perform WHS at request of the company.

The views and policies of the management of OHSs can have an influence on the capabilities, opportunities and motivation of OPs to perform WHS [11, 12]. The majority of the board members did not have a policy to provide OPs with training or instructions related to performing WHS. Horppu et al. (2017) investigated the behaviour of OPs and found that a lack of knowledge and skills can be considered a barrier to performing a desired behaviour [17]. Lugtenberg et al. (2016) also found a lack of knowledge to be a perceived barrier for Dutch OPs to adhere to a guideline [18]. A study of Olsen et al. (2014) showed that physicians play an important role in the implementation of an occupational health and safety programme by influencing the employers [19]. The lack of instructions for OPs can therefore be seen as a negative influence on the capabilities of the OP to perform WHS. No literature on the training needs of OPs in the Netherlands has been found. Persechino et al. (2015) found that Italian OPs regard practical aspects of health surveillance and risk assessment the most important in a training to update their knowledge and skills [20]. Assuming that improving the knowledge and skills of OP on WHS by instructions or training will improve the performance of WHS by the OP, it is important that the managements of the OHSs support the OPs with training in and instructions about the practical aspects of WHS.

Part of the board members reported that WHS is time-consuming. Some board members even mentioned that it will not be possible to perform WHS, as OPs’ schedules are already filled with other activities. This indicates that the infrastructural environment of the OHS is currently often not adapted to the performance of WHS, which can be considered a negative influence on the opportunities for the performance of WHS by OPs. De Brouwer et al. (2017) found that OPs’ activities in the Netherlands mainly focuss on return to work, which leaves them little time to conduct preventive activities [21]. A change in occupational health practice towards a more preventive working environment is needed to improve the performance of WHS.

The contradiction found between the views on WHS – which were partly positive – and the policies adopted, which in general were not actively promoting WHS, may arise because OHSs have to sell their services to employers, who have to pay for it [3, 9]. It appeared from the interviews that opportunities at companies (i.e. employers) to implement WHS were not always present. Some board members mentioned, for instance, that they adopted a passive strategy of only performing WHS upon request, because clients do not see the added value of it and do not respond positively when WHS is recommended, even though there is evidence that WHS can prevent work-related diseases and improve work functioning, and has financial benefits for the employer [5, 6]. This is remarkable as it is the employers’ legal obligation to periodically offer WHS to workers [4]. One of the goals of occupational health care is prevention of work-related diseases [8]. Therefore, OHSs should stimulate the employers to oblige their legal obligations. This sense of urgency seems to be, however, lacking in the approach of a considerable part of the interviewed managers of the OHS.

### Strengths and limitations

The board members of the OHSs were randomly selected from a list with OHSs. This led to the sample containing a large variety of types of OHSs. Assuming that this variety is representative of the variety of OHSs in the Netherlands, the results are applicable for Dutch OHSs in general. This can be considered a strength.

At international level, the European Union Directive recommends to provide workers access to health surveillance, aimed at prevention of work-related and occupational diseases [3]. However, given the specific context of occupational health care in the Netherlands, the current findings may not be generalizable to an international level. This can be considered a limitation of the present study.

Another possible limitation of the study is that it may have been influenced by socially desirable responses [22]. As the invitation for the interview stated that WHS is an important preventive activity, the board members could have felt the pressure to mention that WHS is indeed an important activity. However, board members were open and honest about their policy on WHS, which in almost all cases was not conducive to the performance of WHS. This indicates that the statements about the importance of WHS in the invitation may not have had a strong influence on the answers of the board members.

### Future research

The results of the present study indicate that the current strategy and policy of the managements of the majority of OHSs in the Netherlands are not conducive to the performance of WHS. Future studies should investigate which interventions are most appropriate and effective in order to improve the performance of WHS.

The results of the interviews do not give us information about OPs’ perspective on factors that influence the performance of WHS. In order to improve the performance of WHS, we have started a longitudinal study with OPs, to investigate the needs, barriers, limitations and prerequisites related to performing WHS. The results will be used to develop appropriate intervention strategies to improve the performance of WHS by OPs.

## Conclusion

The present study investigated the view and policy of the managements of OHSs on the performance of WHS. The mission statements and attitudes towards WHS indicated both positive and negative views on WHS. In general, the policy of the managements of OHSs can be considered as not facilitating to the performance of WHS. Managements of OHSs were not providing OPs with any instructions related to WHS, and adopted policies were not stimulating companies to perform WHS. Even though the board members’ mission statements and views on WHS suggest that board members of OHSs are positive about WHS, in practice the policy is in general not conducive to the performance of WHS. A change in the policy of OHSs towards a more preventive approach is needed to improve the performance of WHS.

## Data Availability

The datasets generated and analysed during the current study are not publicly available due to possible recognition of the participants, but are available from the corresponding author upon reasonable request.

## Appendix

### The interview guide used in the semi-structured interviews

#### GENERAL

Which preventive activities are carried out by the occupational health service?

What is the mission statement of the occupational health service?

In what way does the implementation of preventive activities contribute to the realization of the mission statement?

What is the vision statement of the occupational health service?

#### WORKERS’ HEALTH SURVEILLANCE

What is the view of the occupational health service on workers’ health surveillance?

What is the current strategy of the occupational health service regarding workers’ health surveillance?

What is the current policy of the occupational health service on workers’ health surveillance?

## Abbreviations

BCB: Behaviour change ball
OHS: Occupational health service
OP: Occupational physician
WHS: Workers’ health surveillance
